# Imaging cellular structures in super-resolution with SIM, STED and Localisation Microscopy: A practical comparison

**DOI:** 10.1038/srep27290

**Published:** 2016-06-06

**Authors:** Eva Wegel, Antonia Göhler, B. Christoffer Lagerholm, Alan Wainman, Stephan Uphoff, Rainer Kaufmann, Ian M. Dobbie

**Affiliations:** 1 Micron Oxford Advanced Imaging Unit, Department of Biochemistry, University of Oxford, South Parks Road, Oxford OX1 3QU, United Kingdom; 2 Wolfson Imaging Centre Oxford, Weatherall Institute of Molecular Medicine, University of Oxford, Headley Way, Oxford OX3 9DS, United Kingdom; 3 Sir William Dunn School of Pathology, University of Oxford, South Parks Road, Oxford OX1 3RE, United Kingdom; 4 Department of Biochemistry, University of Oxford, South Parks Road, Oxford OX1 3QU, United Kingdom; 5 Division of Structural Biology, Wellcome Trust Centre for Human Genetics, University of Oxford, Roosevelt Drive, Oxford OX3 7BN, United Kingdom

## Abstract

Many biological questions require fluorescence microscopy with a resolution beyond the diffraction limit of light. Super-resolution methods such as Structured Illumination Microscopy (SIM), STimulated Emission Depletion (STED) microscopy and Single Molecule Localisation Microscopy (SMLM) enable an increase in image resolution beyond the classical diffraction-limit. Here, we compare the individual strengths and weaknesses of each technique by imaging a variety of different subcellular structures in fixed cells. We chose examples ranging from well separated vesicles to densely packed three dimensional filaments. We used quantitative and correlative analyses to assess the performance of SIM, STED and SMLM with the aim of establishing a rough guideline regarding the suitability for typical applications and to highlight pitfalls associated with the different techniques.

Classically the resolution of the light microscope is limited by Abbe’s Law to 200–250 nm in the lateral and 500–700 nm in the axial direction. In the last twenty years different techniques were developed to overcome this diffraction limit. The most commonly used super-resolution techniques are STED, SIM and SMLM all of which have been commercialized in the last few years (for detailed reviews of the different techniques see^1^,^2^,^3^).

In Structured Illumination Microscopy (SIM)^4^,^5^,^6^ an adapted wide-field microscope setup uses patterned illumination, usually stripes, to excite the sample. The emitted fluorescence is then recorded for a range of stripe positions and orientations. The interaction between the excitation pattern and the sample produces moiré fringes, allowing capture of high frequency information at lower spatial frequencies. In Fourier space the information from multiple images is computationally separated into low frequency and high frequency information. The separated information is then moved to its correct position and recombined to produce an approximately two-fold increase in resolution in two or three dimensions. In this paper we exclusively use 3D-SIM, which doubles the resolution in all three dimensions. Advances in the technique such as non-linear or saturated SIM are capable of further increasing the resolution but are so far restricted to bespoke setups^7^,^8^,^9^. Commercial solutions for SIM microscopy are available from for example GE Healthcare (Deltavision OMX), Zeiss (Elyra S1), and Nikon (N-SIM).

In STimulated Emission Depletion (STED) a diffraction-limited spot is excited at one wavelength while a super-imposed, red-shifted, second laser beam, projected to a donut-shape, depletes almost all emission laterally leaving only a central focal spot with a dimension below the diffraction limit^10^,^11^. The size of the centre of the focal spot and hence the resolution can be tuned by changing the intensity of the depletion laser such that a lateral resolution of less than 50 nm can be achieved by STED on a commercial system. Initial realisations of STED microscopy utilised pulsed red to far red laser excitation combined with pulsed near infrared laser depletion^12^. A more recent development is time-gated STED (gSTED), which utilises pulsed excitation combined with continuous wave (CW) laser depletion and time-gated detection^13^. Commercial STED solutions are available from Leica (Leica TCS SP8 STED 3X) and Abberior Instruments, including options for gated (CW STED lasers) STED and pulsed STED as well as options for 2D and 3D STED.

Single Molecule Localisation Microscopy (SMLM) relies on the stochastic switching of fluorescent molecules between a bright and a dark state. Having only a few molecules in a fluorescent state at any time allows the location of each molecule to be individually determined with high precision. By taking a few thousand to tens of thousands of images each with a different subset of fluorescent molecules, the position information of the fluorophores can then be used to reconstruct an image with a resolution that mainly depends on the number of detected photons^14^. A resolution in the range of 50 nm can routinely be achieved, further optimisation allows reaching down to the 10 nm range^15^. SMLM is often performed in a TIRF set-up with an optical sectioning better than 200 nm and extremely low background. The disadvantage is that only molecules very close to the coverslip can be detected. A wide-field set-up is also possible at the expense of higher background and no optical sectioning. Axial resolution improvement can be achieved in three ways: by distorting the point spread function (PSF) in the z direction such that the asymmetry of the PSF then correlates with the z position of the emitter and allows a more precise extraction of the z position^16^,^17^, by the simultaneous detection of two object planes^18^, or an interferometric approach^19^. In general SMLM can be performed using either special photo-switchable or photo-activatable fluorophores/dye pairs^20^,^21^,^22^ or standard fluorescent proteins and organic dyes^23^,^24^,^25^. Commercial solutions are available from Leica (Leica GSD), Nikon (N-STORM), Zeiss (Zeiss Elyra P1) and Bruker (Vutara 350).

Theoretical discussions of the individual strengths of super-resolution techniques have been published^1^,^2^,^3^,^26^ but a practical comparison of the performance of all three techniques has been missing to date. Here we imaged the same kinds of samples with SIM, STED and SMLM. For the comparisons we used microtubules, centrioles and vesicles as examples for densely labelled but well separated structures and actin filaments in COS7 cells as an example for densely packed three dimensional structures while the trans-Golgi network represents an intermediate type of cellular structures. As well as the realistically achievable resolution we assessed the suitability of each technique for the different biological examples. We point out issues and challenges regarding sample preparation, multi-colour imaging and image reconstruction as well as complexity and cost of the different systems.

## Results

For the super-resolution comparisons we chose a range of biological structures that vary in shape and complexity in all three dimensions, i.e. strongly fluorescent 25 nm filaments of varying density in the cell (microtubules^27^), 9 nm filaments in two close layers of fine meshwork (actin^28^), hollow barrels (centriole walls), vesicles and finally tubules in a complicated 3D arrangement (trans-Golgi network). The cell components were labelled with primary and secondary IgG antibodies with the exception of F-actin, which was labelled with phalloidin directly coupled to an Alexa Fluor dye. Z-stacks were acquired for SIM, which is the standard and only SIM acquisition mode possible with the microscope we used. In order to achieve maximum lateral resolution in the single colour experiments single plane STED images were acquired in 2D with maximum STED power thus sacrificing any possible resolution improvement in the axial direction. Two-colour STED experiments were only performed at half maximum STED laser power, which was judged to be a reasonable compromise between lateral resolution enhancement, signal-to-noise and cross-talk between imaging channels. The SMLM experiments were carried out in TIRF mode where the cell components were sufficiently close to the coverslip as this results in a minimal optical section thickness and dramatically cuts down on out of focus light and thereby improves the signal to noise ratio. The centrioles are too far from the coverslip for TIRF imaging, but are also isolated structures with little out of focus blur and were imaged in wide-field mode, like-wise, the Golgi in the two-colour experiments were also imaged in widefield mode because of too much nonspecfic signal on the coverslip.

**Figure 1:**
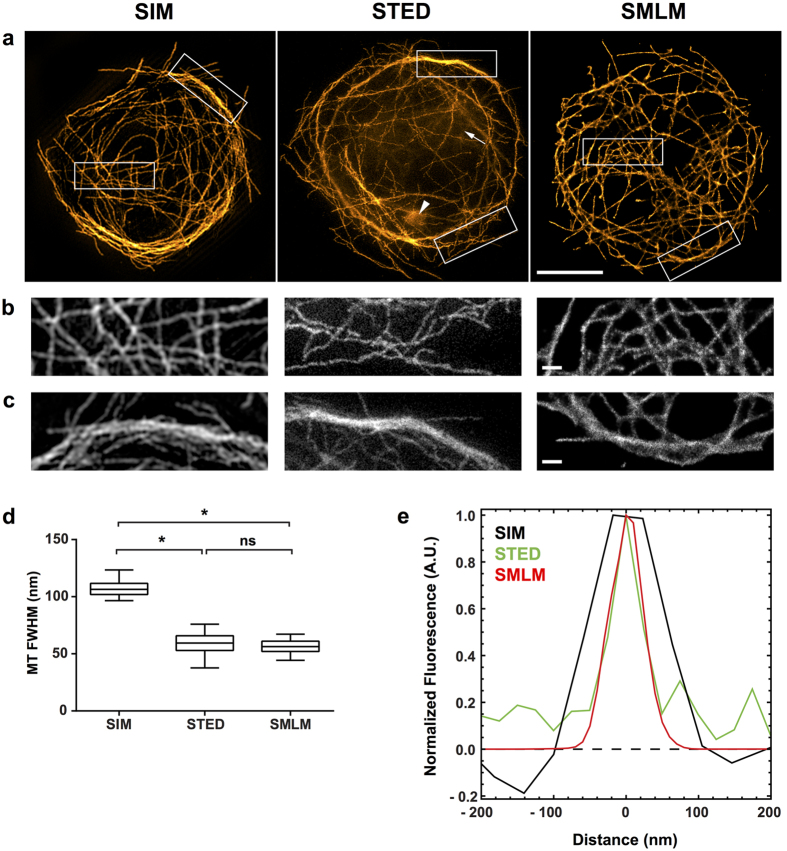
Microtubules in Drosophila macrophages. Microtubules were detected with primary and secondary antibodies, the latter coupled to Alexa Fluor 488 for SIM and STED and Alexa Fluor 647 for SMLM. 100% available depletion laser power was used for STED. (**a**) Single optical section through the microtubule network close to the coverslip. Scale bar, 5 μm. The boxed areas are magnified in (**b**,**c**). In the STED image out of focus blur (arrow) and an artefact caused by high laser intensity (arrow head) are visible. (**b**) Region showing well separated microtubules. Scale bar, 0.5 μm. (**c**) Region with microtubule bundles. Scale bar, 0.5 μm (**d**) Results of FWHM measurements of 50 microtubules per super-resolution technique. (**e**) Representative normalised line profiles for each super-resolution technique. Close to the microtubule, intensity values for SIM can dip into negative values, which is an artefact caused by the SIM reconstruction.

Microtubules were imaged in spread *Drosophila* macrophages (Fig. 1). The cells resemble fried eggs and their microtubules are mostly distributed around the central bulge, which is 3 to 4 μm high and contains the nucleus and the bulk of the cytoplasm. All three techniques resolved well separated microtubules (Fig. 1b) but not microtubule bundles (Fig. 1c). An analysis of 50 microtubules for each technique showed that there was no statistically significant difference between SMLM and STED in the measured FWHM (±s.e.m) of microtubules (mean values 56.3 (±0.78) nm and 58.8 (±1.2) nm respectively). The mean value for SIM was, as expected, in the region of half the diffraction limit with 107 (±0.85) nm (the conventional limit of resolution is ~220 nm in this system, Fig. 1d). The STED image contains some blur due to noise and out of focus signal and an artefact caused by high STED laser power (Fig. 1a). It also has poorer image contrast than the SIM and SMLM images (Fig. 1a–c). As shown in Table 1, STED data for both microtubules and the centrioles below have a lower signal to noise ratio within the structure and a lower signal to background ratio than their SIM and SMLM counterparts.

**Figure 2:**
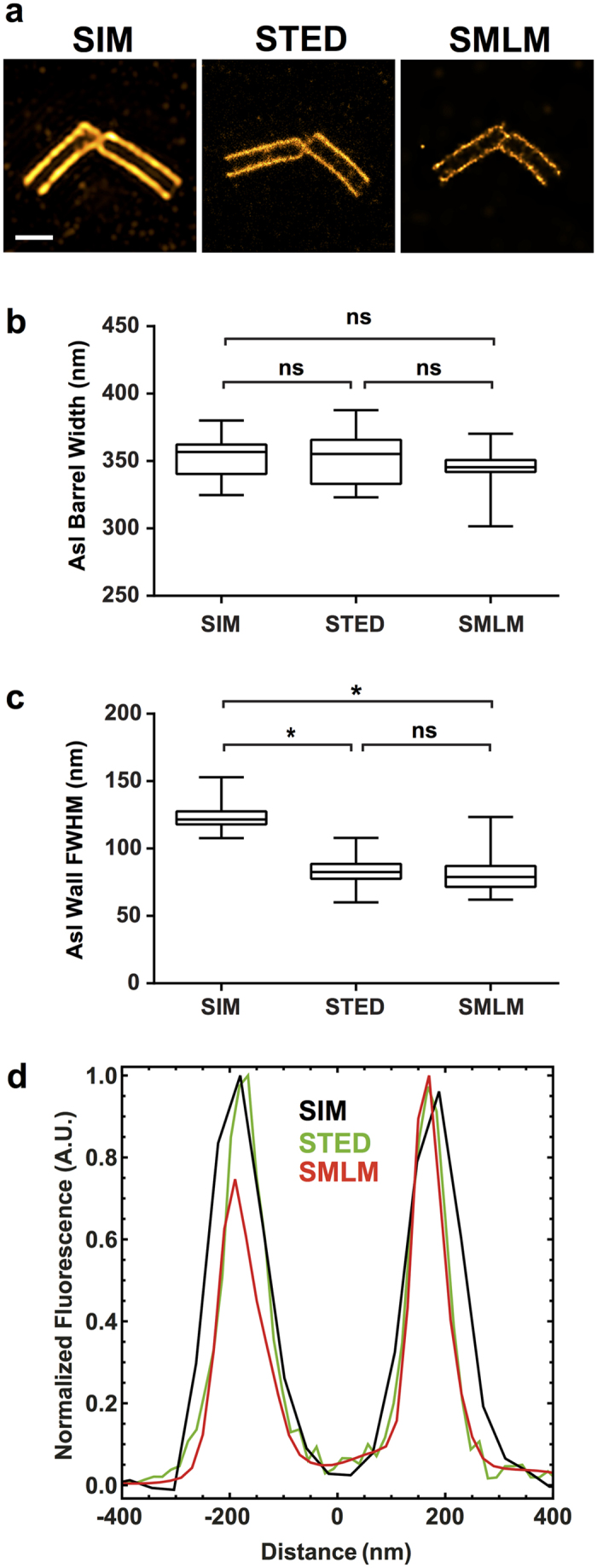
Centrioles in Drosophila primary spermatocytes. Asterless (Asl) localisation in an orthogonal centriole pair was detected with primary and secondary antibodies, the latter coupled to Alexa Fluor 488 for SIM, STED and SMLM. 100% available depletion laser power was used for STED. Scale bar, 0.5 μm. (**a**) Single optical section through the centre of the centrioles. (**b**) Measurements of the barrel widths of 30 centrioles per super-resolution technique. (**c**) FWHM measurements of 60 centriole walls per super-resolution technique. (**d**) Representative normalised line profiles for each super-resolution technique.

The centrioles in *Drosophila* primary spermatocytes form unusually long barrels and provide an excellent example of a highly labelled structure with defined dimensions in two size ranges, one below the diffraction limit and one above. We visualised them using Asterless (Asl), a component of the *Drosophila* centriole wall^29^,^30^ and performed two measurements for each imaging technique: 1) the barrel width (N = 30), and 2) the FWHM of the Asl localisation signal in the centriole wall (N = 60) (Fig. 2). There was no statistically significant difference in barrel widths between the different imaging techniques where the mean values (±s.e.m) were for SIM: 353 (±2.8) nm, STED: 352 (±3.3) nm, and SMLM: 344 (±2.7) nm (Fig. 2b). There was also no statistically significant difference between SMLM and STED in the measured FWHM of the Asl localisation signal (mean values 81.4 (±1.7) nm and 83.5 (±1.9) nm respectively), while the FWHM measured with SIM was significantly broader (123 (±1.2) nm, Fig. 2c).

**Figure 3:**
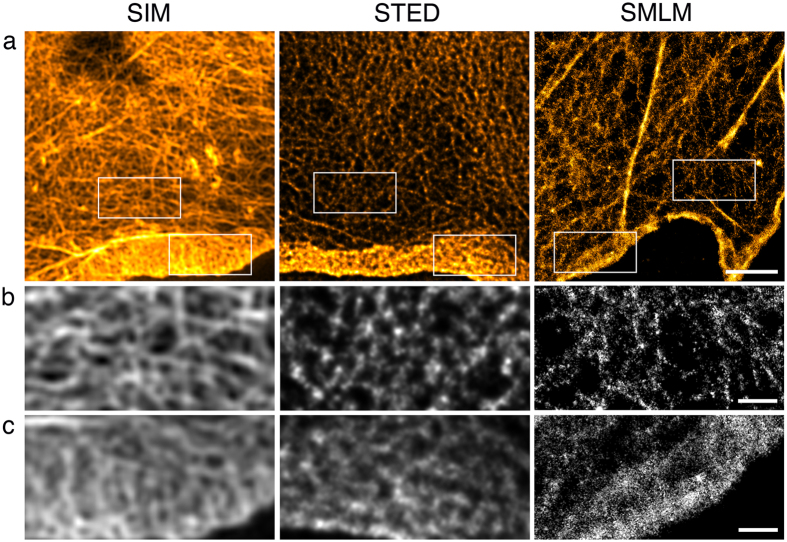
Actin in COS7 cells. Actin was detected with phalloidin coupled to Alexa Fluor 488 for SIM and STED and Alexa Fluor 647 for SMLM. 100% available depletion laser power was used for STED. (**a**) Single optical section through the cell periphery. Boxed areas depict parts of the lamella (**b**) and the lamellipodium (**c**). The STED image has been 2D deconvolved. Scale bar, 2 μm. (**b**) Fine structure of the lamella. Scale bar, 0.5 μm. (**c**) Fine structure of the lamellipodium. Scale bar, 0.5 μm.

F-actin can form a variety of very dense, three-dimensional meshworks within cells. Here we imaged F-actin close to the coverslip in well-spread COS7 cells (Fig. 3). The actin meshwork in the lamella was best resolved in SIM (Fig. 3b). STED images of this dense structure generally suffered from low contrast (Supplementary Fig. S1). Consequently the STED example for this structure was deconvolved to increase contrast and reduce out-of-focus blur. However, this did not improve meshwork resolution to the level of the SIM images. In our hands SMLM resolves stress fibres well but thin filaments are often dotty and uneven and only visible in some regions. Actin filaments within the lamellipodium, which has a denser meshwork than the lamella^31^, were not well resolved in any technique.

**Figure 4:**
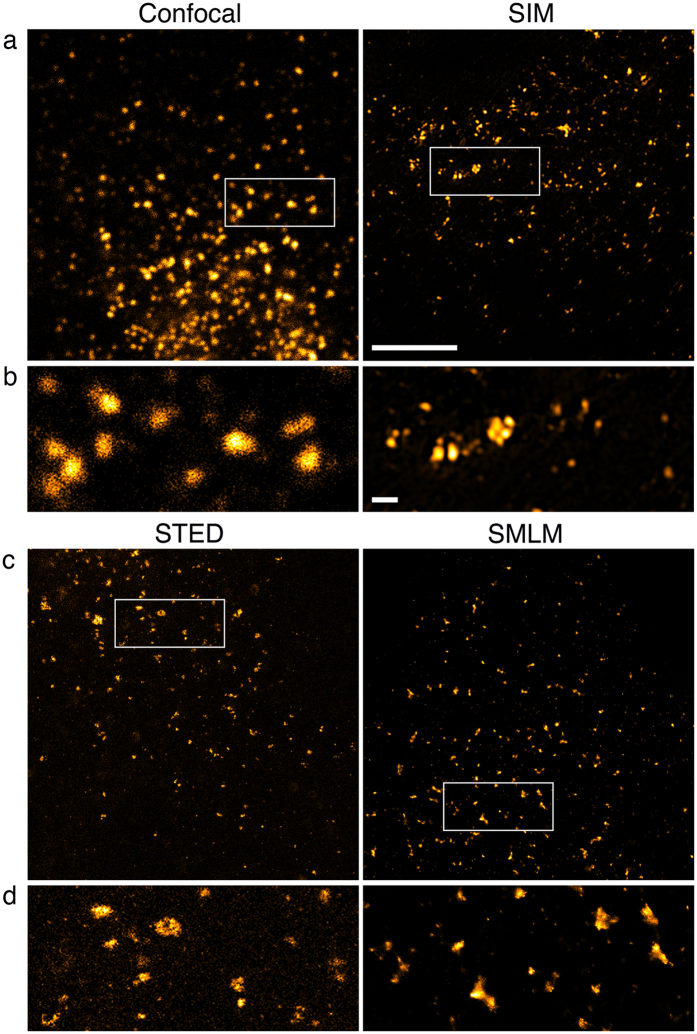
Transport vesicles in COS7 cells. Sec31A was detected with primary and secondary antibodies, the latter coupled to Alexa Fluor 488 for confocal imaging, SIM and STED and Alexa Fluor 647 for SMLM. 100% available depletion laser power was used for STED. (**a,c**) Overview of vesicles close to the nucleus in a single optical section. Boxed areas are magnified in (**b,d**). Scale bar, 5 μm. (**b,d**) Vesicles of various sizes with a little out of focus background visible as weak, small foci in STED. Scale bar, 0.5 μm.

Human Sec31A is a component of the coat protein complex II (COPII), which is found on vesicles that mediate ER to Golgi transport^32^. COPII vesicles in COS7 cells were distributed most densely close to the nucleus presumably in the vicinity of the Golgi apparatus (Fig. 4). The three super-resolution techniques revealed a wide range of vesicle sizes that were not apparent in the confocal data included for comparison (Fig. 4a). From SIM to STED to SMLM the amount of detail visible in the distribution of Sec31A increased. Some out of focus blur was visible in the densest regions of both the confocal and the STED images (Fig. 4a,d).

**Figure 5:**
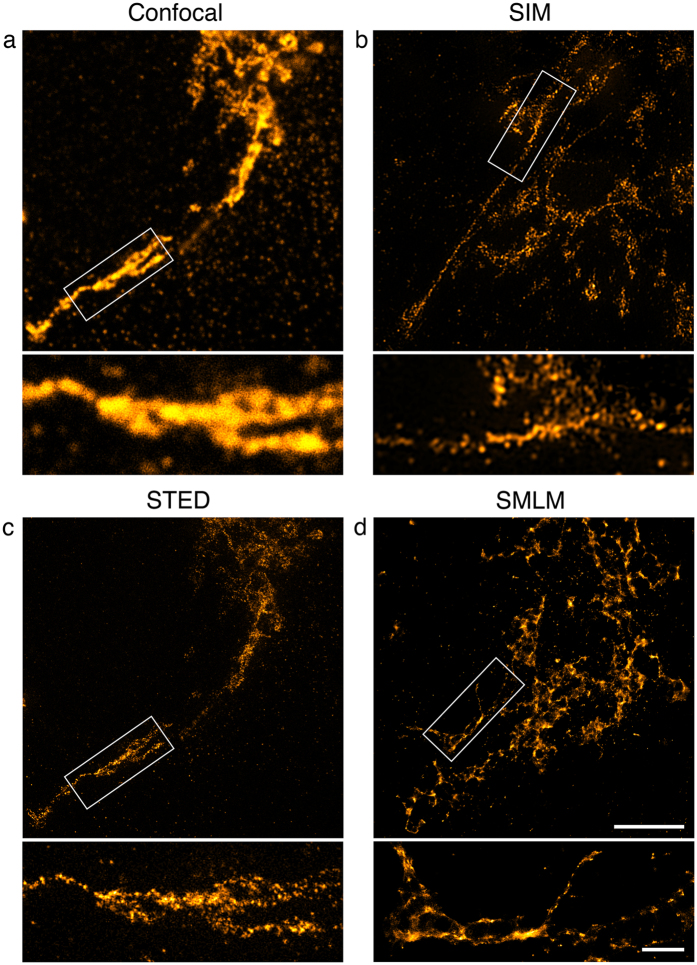
Trans-Golgi network in COS7 cells. TGN46 distribution was detected with primary and secondary antibodies, the latter coupled to Alexa Fluor 488 for confocal imaging (**a**), SIM (**b**) and STED (**c**) and Alexa Fluor 647 for SMLM (**d**). 100% available depletion laser power was used for STED. Shown are single optical sections through part of the TGN. Scale bar, 5 µm. Boxed areas are magnified underneath. Scale bar, 1 µm.

Human TGN46 is an integral membrane protein and a marker for the trans-Golgi network (TGN) cycling between the TGN and the cell membrane^33^,^34^. In addition to the complicated tubular network the antibody also labelled vesicles close to the Golgi (Fig. 5). Vesicles and TGN structures were resolved in all three techniques and look comparable. TGN46 signal was not continuous even in confocal images (Fig. 5a) and dotty distribution of the label was particularly obvious in SIM and STED and less so in SMLM (Fig. 5b–d).

**Figure 6:**
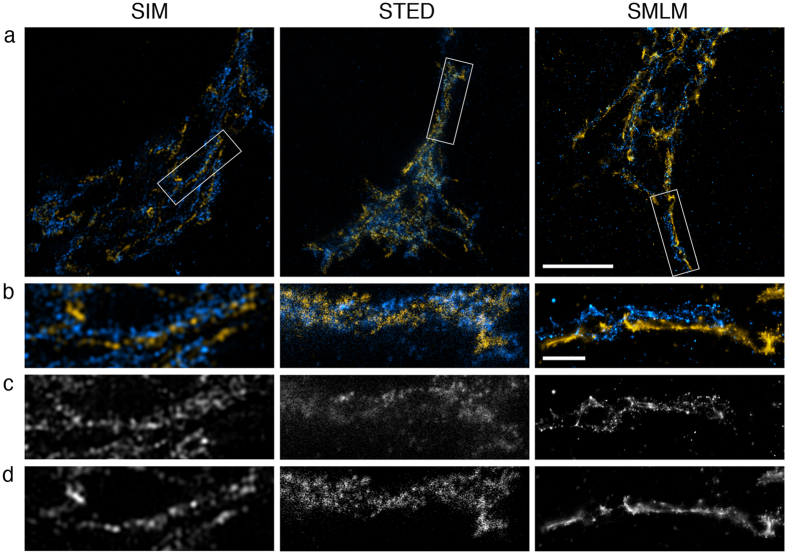
Trans- and cis-Golgi in COS7 cells. TGN46 distribution (blue) was detected with primary and secondary antibodies, the latter coupled to Alexa Fluor 488 for SIM and STED and Alexa Fluor 647 for SMLM. GM130 distribution (yellow) was detected with primary and secondary antibodies, the latter coupled to Alexa 594 for SIM, TMR for STED and Alexa 532 for SMLM. 50% available depletion laser power was used for STED. (**a**) Single optical section through part of the Golgi. Boxed areas are magnified in (**b–d**). Scale bar, 5 μm. (**b**) Fine structure of the cis- and trans-Golgi. Scale bar, 1 μm. (**c**) TGN46 localisation. (**d**) GM130 localisation.

In the final experiment we compared two-colour imaging in all three techniques. We chose GM130 as a peripheral cytoplasmic protein that is tightly bound to membranes of the cis Golgi network and cis Golgi cisternae^35^ and TGN46 as the trans Golgi network marker. Both label distinct Golgi compartments that do not overlap. In SIM and SMLM images double-labelled with GM130 and TGN46 the two markers were in close proximity but had very little overlap (Fig. 6a,b). In STED images out of focus blur was apparent, which made it hard to see a separation between the two parts of the Golgi.

## Discussion

We have imaged a variety of biological structures to compare the three commercially available super-resolution techniques SIM, STED and SMLM, and to highlight the strengths and weaknesses of each technique for the different test cases. Table 2 summarises the results and categorizes them to evaluate the achievable resolution, the image quality of the different structure types and the overall performance of the technique with regards to image reconstruction, sample preparation, multi-colour imaging and the system’s complexity itself.

The achievable resolution of linear SIM (as used here) is constrained to a hard limit of approx. 110 nm in the green (e.g. see Fig. 1d,e). Fine detail below this threshold will appear at this size. SIM gives good results with widely spaced structures (Figs 1,2 and 4), with dense meshworks (Fig. 3) and signal that extends along the z-axis (Fig. 5) provided there is adequate contrast. For dense three-dimensional networks composed of fine, and therefore relatively weakly labelled, structures such as the actin cytoskeleton, SIM is probably the super-resolution technique of choice unless highly specialist equipment is available. However, none of our methods was able to resolve the underlying structure of 9 nm fibres.

With a good signal to noise ratio unknown structures will be rendered into a credible image. However, low contrast in SIM raw data can lead to reconstruction artefacts with a feature size in the same range as the intended resolution. An adjustable Wiener filter is used in the reconstruction algorithms to filter out noise. If the Wiener filter is set too low for the quality of the raw data then ‘wiggly’ artificial structures appear within the signal and in the background (Supplementary Fig. S2). The Wiener filter setting also affects the resolution of the SIM image^36^. Other artefacts are caused by mismatches between the refractive index of the immersion oil and the embedding medium. The reconstruction algorithms are specific to the commercial systems they come with; an open source algorithm has just been published^37^ but there are, as yet, no standard comparisons between reconstruction algorithms although tools such as SIMcheck^38^ can help assess reconstruction quality.

SIM allows the use of the same chemical dyes as in wide-field and confocal microscopy and multi-colour SIM z-stacks are routine, although in practice the best resolution is often achieved with 488 nm excitation. Mounting conditions are the same as for conventional immunostaining meaning moving from conventional imaging to SIM super-resolution imaging is relatively easy.

The specified lateral resolution of the commercial STED microscope used here is 50 nm, although custom-built setups have achieved significantly higher resolutions^39^. In the examples presented, the accessible resolution is best evidenced by the FWHM measurements of the microtubule filaments in Fig. 1. Uniquely among the three tested techniques, the super-resolution image in STED is achieved by optics alone and the resolution can be tuned by changing the power of the STED laser (Supplementary Fig. S3). In contrast, extensive computational processing as required for both SIM and SMLM is not necessary thus removing the possibility of image reconstruction artefacts. However, even though STED images do not *per se* require computational processing, image contrast is frequently low as seen in Figs 1, 2, 3, 4, 5, 6. Image contrast can be improved by a variety of contrast enhancement techniques albeit, in our case, with a loss in absolute resolution (Supplementary Fig. S4). The need for contrast enhancement is particularly clear in very dense samples as is the case for the actin meshwork in Fig. 3. Alternatives to computational processing include reducing the depletion laser power (Supplementary Fig. S3), extensive line or frame averaging, or both. In the end, it is often best to compare different acquisition and post-processing strategies carefully. A potential drawback in STED is that the powers of the STED lasers far exceed the laser power that is required in a conventional laser scanning confocal. Thus, caution is always advisable in the use of STED. In particular, care must be taken to ensure that the specimen has minimal absorption at the STED wavelengths that are used. Dye selection and mounting conditions are important to produce good STED images^40^.

The biggest strength of SMLM is a relatively simple optical setup while still achieving diffraction unlimited resolution. As our examples show a lateral resolution of 50 nm can routinely be achieved (Fig. 1) without having to use special fluorophores. The microtubule measurements were carried out on cells that had been immunolabelled with two full-size IgG antibodies, which increased the size of the microtubules to around 50 nm. The use of one directly labelled antibody or (Fab)_2_ fragments as secondary antibodies would have allowed a more stringent comparison between the achievable resolution on a commercial STED microscope and an SMLM microscope. Other labelling strategies can enable a resolution down to 10 nm or less^15^. For single colour experiments we used Alexa Fluor 647, which is the most commonly used chemical dye because of its high emitted photon count and convenient switching behaviour^41^.

The actin meshwork (Fig. 3) is an example of structures that are difficult to image with SMLM as evidenced by results produced in other labs^42^,^43^. Very convincing actin images are extremely challenging and have only been published by specialist labs such as Xu *et al*.^15^. Several factors contribute to this. To adequately resolve a structure one needs to sample it (i.e. detect fluorescent molecules) at a rate of more than twice the desired resolution (Nyquist-Shannon theorem). This requires very high label densities, which can only be achieved if the detected protein forms dense clusters, coils or other macromolecular structures (see also^44^). Polyclonal antibodies, which recognise several epitopes, can be helpful here provided their binding is specific to the target protein. The fact that actin fibres are composed of two helical filaments, whereas microtubules are made up of 13 protofilaments of heterodimers, means that there are roughly six times as many binding sites per unit length on microtubules compared to actin. This makes it very difficult to achieve a phalloidin density that is high enough to resolve the structure. Another factor is background label. Detectable amounts of target protein diffusing through the cytoplasm will contribute to specific background, while high concentrations of antibody can lead to nonspecific binding, both resulting in localisations, i.e. noise, outside the structure. A third factor is out of focus fluorescence in three-dimensional structures like the actin meshwork. The increased background intensity reduces the localisation precision, which together with signal density determines structural resolution. This highlights the importance of labelling quality and also the choice of fluorophores and imaging conditions (e.g. on-off-ratio, irreversible bleaching). Although most fluorophores are in principle suitable for SMLM, multi-colour imaging requires careful selection and matching of fluorophores. Choosing buffer and mounting conditions that are suitable for all fluorophores is of particular importance (for guidelines see^41^,^45^) and often a compromise is required. Here the dye pair Alexa 647/Alexa 532 worked best (Fig. 6) although we detected four times fewer photons in the red channel compared to single colour imaging of Alexa 647 under similar conditions (Fig. 5). This highlights the compromised buffer conditions we used to enable good blinking in both dyes. In general high laser intensities are required if the imaging is done in hard-setting mounting media (e.g. Mowiol, Immumount, Prolong Gold)^23^,^46^. Poor imaging conditions also have an impact on the algorithms used for SMLM image reconstruction. Raw data, which contain background signal, high signal density, unsuitable switching parameters or sample drift, present additional challenges for the reconstruction software. If not considered, this can lead to false positive, or false negative, localisations (supplementary Fig. S5, comparison of the same raw data analysed with two different algorithms), and thus to artefacts in the reconstructed SMLM image. Currently more than 50 algorithms exist, but not all are freely available^47^,^48^.

Proper sample preparation is important for all three techniques but the details and constraints can vary considerably. Good preservation of subcellular structure is paramount in super-resolution microscopy and, with few exceptions, samples have to be fixed with a crosslinking fixative (commonly paraformaldehyde, for a discussion of fixation artefacts see^42^,^49^). In general therefore, methanol, methanol-containing formalin and other precipitating fixatives should be avoided. High labelling density is also very important as discussed above. The higher the desired resolution the higher the labelling density needs to be. SIM, STED and SMLM are all advanced imaging techniques, but the complexity and cost of the imaging systems required differs significantly. SIM and STED require extremely complex optics and control hardware, which is reflected in their price, roughly twice the cost of a fully featured confocal microscope. However, SMLM has relatively simple optical requirements: a widefield or TIRF microscope setup with one or more powerful lasers, and a stage with minimal drift because of the long acquisition times needed. The additional complexity in SMLM comes from the analysis process.

Although SIM, STED and SMLM differ in their fundamental resolution (see Fig. 1e), with lateral resolutions of roughly 110 nm, 55 nm and 55 nm respectively in our hands, the separation of Asl in the centriole barrel is found to be the same in all techniques to an extremely high precision. This clearly demonstrates that imaging resolution is not always the most important factor, issues such as labelling, contrast and ease of sample preparation being significant factors. Additionally, all super-resolution techniques suffer from increasing optical aberrations with increasing imaging depth into the cell. How fast the decline is depends on the aberrations present in the sample such as refractive index mismatch/variation or light scattering. Lastly, acquisition times differ widely between the different techniques. The acquisition of a z-stack in SIM takes up to a few minutes, while acquisition of single STED images ranges from seconds to minutes depending on frame sizes and scan speeds. SMLM movies comprising a few tens of thousands of frames can take up to twenty minutes. The overall number of frames depends on the labelling density, the switching efficiency and the detected number of photons per fluorophore. Integration times should be in the range of the on-times of the fluorophore.

In summary, the techniques presented here significantly enhance the resolution possible in fluorescence imaging, with SIM providing twice the resolution in two or three dimensions, whereas both STED and SMLM provide diffraction unlimited resolution. However, all the techniques require significant experience and expertise to use. With this in mind it is advisable to utilise an imaging facility or specialist lab with the required expertise to get the most from super-resolution imaging experiments. It is also important to match the technique used to the experimental aims and to properly process the acquired data, including adequate control experiments, as even small errors in image processing can significantly affect your results. The development of tool kits for STED and SMLM dedicated to checking the quality of raw and where applicable reconstructed data similar to the existing one for SIM^38^ might enable less experienced users to reliably achieve better results. Super-resolution imaging users should also carefully consider that as imaging resolution increases true structural resolution is significantly affected by factors such as label density and label size.

## Materials and Methods

### Preparation of cells for immunostaining

*Drosophila* macrophages were prepared from third instar larvae according to^50^. They were allowed to settle in Schneider’s insect medium (Sigma, S0146) on glass coverslips for two h at 25 °C. We used Marienfeld high precision coverslips no. 1.5H (UK distributor Cellpath) for all experiments. Cells were pre-extracted for 5 s in PBS, 0.1% NP40 (Igepal Ca-630, Sigma-Aldrich, I3021) before fixation in 1% methanol free paraformaldehyde (Polysciences, 18814) in PBS for 50 min. They were then permeabilised for 10 min in 0.1% Triton X-100 (Sigma-Aldrich, X100) in PBS and washed in PBS. *Drosophila* primary spermatocytes were prepared from testes according to^51^,^52^. Briefly, testes were fixed in 3.7% methanol containing formaldehyde (VWR, 20909.290) for 30 min, treated with 45% acetic acid for 45 seconds and 60% acetic acid for 3 min, snap frozen in liquid nitrogen and post fixed in in ethanol for 15 min. They were then permeabilised and washed as above. The Asl antibody signal obtained after this fixation was similar to Asl-GFP imaged *in vivo* using SIM (Supplementary Fig. S6).

COS7 cells were grown for 24 to 48 h on glass coverslips to 80% confluence in high glucose Dulbecco’s modified Eagle’s medium (DMEM, Life Technologies, 31966-21) supplemented with 10% (v/v) FBS (Life Technologies, 10500). They were fixed in 3% methanol free paraformaldehyde in PBS for 15 min at 37 °C and then permeabilised for 7 min in 0.1% Triton X-100 in PBS before washes in PBS.

### Immunostaining

*Drosophila* macrophages and COS7 cells were blocked in PBS, 0.1% Tween 20 (Sigma, P9416), 3% BSA (Sigma, A3059) for 30 min. Primary and secondary antibody incubation in blocking solution was carried out for one hour with wash steps in PBS, 0.1% Tween 20 after each incubation. The following primary antibodies were used: monoclonal mouse anti α-tubulin 1:1000 (Sigma-Aldrich T6199, clone DM1A), polyclonal sheep anti human TGN46 1:1000 (AbD Serotec, AHP500G), monoclonal mouse anti human GM130 1:1000 (BD Biosciences, 610823), monoclonal mouse anti human Sec31A 1:1000 (BD Biosciences, 612350). Actin was detected with Phalloidin conjugated to Alexa Fluor 488 (Life Technologies, A12379) at a final concentration of 0.5 Units per 200 μL or to Alexa 647 (New England Biolabs, 8940S) at a final concentration of 4 Units per 200 μL. Phalloidin incubation was for 45 min for SMLM and for one hour for all other experiments in blocking solution. *Drosophila* Asterless was detected using guinea pig anti Asl 1:1000^52^. Secondary antibodies used at concentrations of 1:500 or 1:800 were as follows: Donkey anti mouse or donkey anti sheep conjugated to Alexa Fluor 488 or Alexa 647 (Life Technologies, A21202, A11015, A31571, A21448), goat anti-guinea pig conjugated to Alexa Fluor 488 (Life Technologies, A11073). For SIM, donkey anti mouse and donkey anti sheep conjugated to Alexa Fluor 594 and Alexa Fluor 488 respectively (Life Technologies, A21203, A11015) was used for double-labelling experiments. For STED, goat anti mouse and donkey anti sheep conjugated to tetramethylrhodamine (TMR) and Alexa Fluor 488 (Life Technologies, T2762, A11015) respectively was used. For SMLM double-labelling experiments, donkey anti sheep and goat anti mouse conjugated to Alexa Fluor 647 and Alexa Fluor 532 (Life Technologies, A21448, A11002) respectively was used. For SIM and STED experiments COS7 cells and *Drosophila* macrophages were embedded in Prolong Gold (Life Technologies, P36930) and the coverslips sealed after 16 to 20 h. *Drosophila* testes preparations were embedded in Vectashield (Vector Laboratories, H-1000) for SIM imaging and in Mowiol 4–88 (Polysciences, 17951) supplemented with 2.5% (v/v) DABCO (Sigma-Aldrich, 290734) for STED and SMLM experiments. For SMLM experiments requiring switching buffer coverslips were either imaged directly or kept in PBS overnight and imaged the following day.

### Microscopy

For SIM, cells were imaged with a 60x, NA 1.42 oil objective on a Deltavision OMX V3 Blaze (GE) equipped with 488 nm and 592 nm lasers. Spherical aberration was minimised by choosing an immersion oil with a refractive index giving symmetrical point spread functions and image stacks of several μm thickness were taken with 0.125 μm z-steps and 15 images (three angles and five phases per angle) per z-section and a 3D structured illumination with stripe separation of 213 nm and 238 nm at 488 nm and 594 nm respectively. It should be noted that the OMX microscope used in this paper does not allow the collection of 2D SIM images. Image stacks were reconstructed in Deltavision softWoRx 6.1.1 software with a Wiener filter of 0.002 using wavelength specific experimentally determined OTF functions as described in^6^. This leads to a halving of the pixel size and a doubling of the X and Y direction pixel number (i.e. four times as many pixels), but no change in Z-steps in the image stacks.

As the OMX system uses separate cameras for each colour, image alignment is critical. For two-colour experiments, reference z-stacks of 200 nm TetraSpeck beads (Life Technologies, T7280) were aligned with custom rigid body alignment routines correcting for x, y and z shifts, magnification and rotation differences between channels. The transformations were then applied to align the two-channel images.

For STED, cells were imaged with a 100x, NA 1.4 oil objective on a Leica SP8 gated STED microscope. Alexa Fluor 488 labelled probes were excited with the 488 nm wavelength of a pulsed white light (WL) laser (80 MHz) and depleted with a CW 592 nm STED laser with a typical maximum power of 260–300 mW at the back aperture of the objective (corresponding to ~150 MW/cm^2^ in the focal plane), while TMR labelled probes were excited with the 561 nm wavelength of a WL laser and depleted with a CW 660 nm STED laser with a typical maximum power of 240–280 mW. All images were acquired in 2D STED mode, i.e. with only lateral resolution improvement. Structures in Figs 1, 2, 3, 4, 5 were imaged at settings optimized for a maximum gain in lateral resolution. This corresponded to full depletion laser on an internal Leica GaAsP HyD hybrid detector with a time gate of 1.5 ≤ t_g_ ≤ 6.5 ns. An exception to the above is the data for Supplementary Fig. S3, which was imaged as a function of STED laser power but with the same time gate. The two colour STED data in Fig. 6 were imaged at 50% of maximum power both for TGN46 and GM130 but with equivalent time gates. As shown in Supplementary Fig. S3, the resolution at this setting is about 80 nm.

Deconvolution of STED data was carried out using the STED module in Huygens Professional Deconvolution software (version 14.10; Scientific Volume Imaging). This software contains a theoretical estimation of the STED PSF that is based on values that are calculated from the metadata of the acquired image. For the deconvolved images presented here we have used the calculated Huygens default deconvolution settings that were estimated from the metadata except that we used a STED immunity fraction of 5%. A simple alternative to deconvolution solely for the purpose of minimizing noise is to apply a Gaussian 2D kernel with a Gaussian width, σ, to the image data. In our case, we have used the Gaussian Blur filtering function in the Fiji compilation of ImageJ 1.47N (ImageJ version: 2.0.0-rc-19/1.49m, http://imagej.net)^53^ with a Gaussian width of 1 or 2 pixels.

SMLM imaging was performed with three different setups. Using single colour SMLM with a standard mounting medium and a single laser wavelength^23^,^46^, centrioles were imaged on a Deltavision OMX V2 microscope (Applied Precision) equipped with a 100x, NA 1.4 oil objective (UPlanSApo, Olympus), a 488 nm laser and a customized SMLM lightpath with approx. 30 kW/cm^2^ laser intensity as detailed in^54^. 4000 images were taken in wide-field illumination with an integration time of 50 ms. SMLM imaging with switching buffers to enhance single molecule photo-switching^55^ was used for all other single colour experiments and performed on a home-built wide-field microscope equipped with a 100x, NA 1.4 oil objective, a TIRF lens and a 640 nm laser with 1.5–2 kW/cm^2^ laser intensity (for details see Supplementary data STORM setup). For the detection of microtubules, vesicles and trans Golgi, cells were imaged with TIRF illumination to reduce out of focus fluorescence, in the following switching buffer: 27 U/mL glucose oxidase (Sigma, G2133), 402 U/mL catalase (Sigma C100), 10% (w/v) glucose, 100 mM MEA (2-mercaptoethlyamine hydrochloride, Alfa Aesar, A14377) in PBS pH 7.4. 15,000 images were taken with an integration time of 20 ms. Actin was imaged using the same switching buffer as above but with twice the enzyme concentration and at pH 7.7. 30,000 images were taken with an integration time of 30 ms. For two-colour SMLM experiments using switching buffer (10 mM MEA in PBS, pH 7.8), cells were imaged on a Leica GSD microscope equipped with a 160x, NA 1.43 oil objective, a 500 mW 532 nm laser, a 500 mW 642 nm laser and a 30 mW 405 nm laser. Alexa Fluor 647 was imaged first with wide-field illumination and an integration time of 20 ms and Alexa Fluor 532 second, also with an integration time of 20 ms and backpumping into the on-state using the 405 nm laser. 15,000 images per channel were taken with a field of view of 180×180 pixels and 100 nm pixel size. RapidSTORM^56^,^57^ was used to reconstruct single molecule localisations of actin, vesicles, the trans-Golgi network and the two-colour data. For the position determination of the single fluorescent molecules in microtubule and centriole experiments we used fastSPDM^58^, which was adapted to the hardware configuration of the microscope setup. Drift of the microscope stage was corrected based on the SMLM position data. Super-resolution images were generated from the SMLM position data based on nearest neighbour distances to also consider the Nyquist limited resolution^54^,^59^. We compare these two analysis routines on the same raw data in Supplementary Fig. S5 to demonstrate how different analysis routines can produce different artefacts in the reconstructed data.

### Size measurements

For every super-resolution technique we plotted the profile of ten microtubules each from five cells and fitted a Gaussian curve using Fiji open source image processing software (ImageJ version: 2.0.0-rc-19/1.49m, http://imagej.net). For the profile a line width of ca. 165 nm was chosen in order to cover a minimum width of four pixels in SIM images and more in STED and SMLM. Microtubule widths in SIM stacks were measured in the z-section where the microtubule fluoresced most strongly. The full width at half maximum (FWHM) was calculated with the following formula where σ is the standard deviation of the Gaussian:


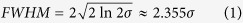


For centriole measurements, intensity line profiles with a line width of ca. 165 nm, were drawn near the end of the centriole barrel and across both centriole barrel walls using Fiji as above. A sum of two Gaussian curves was then fitted to the line profiles in Mathematica v 9.01 (Wolfram). The distance between the walls of each centriole was determined from the distance between the centre of each Gaussian curve and FWHM values were calculated separately from the standard deviations, σ1 and σ2, for each centriole wall as described for the microtubule measurements. Box and whisker plots were generated in Prism v6 (GraphPad). The box in this case extends from the 25^th^ to the 75^th^ percentile of the data and the median value is marked with a line. The whiskers extend from the smallest value to the largest value measured. Statistical significance of all measurements was also evaluated in Prism by using a two-tailed Mann-Whitney test of repeated measures. Statistical significance was considered for P values <0.05.

### Signal to noise measurements

The signal to noise measurements were carried out in Fiji using a 1 pixel freehand line to measure signal intensities on the structures. Six cells with labelled microtubules and 15 centriole pairs were measured for each technique. Background areas close to the structures within every analysed cell were also measured.

Figures were prepared in Fiji using the Orange Hot, Cyan Hot and Yellow Hot look up tables. SIM and STED images were scaled to the same pixel size as the SMLM data with bicubic interpolation. In the SIM images, negative values, which are artefacts caused by the reconstruction algorithm, were set to zero intensity (the mode value). The figures were then compiled in Photoshop and Illustrator (Adobe).

## Additional Information

**How to cite this article**: Wegel, E. *et al*. Imaging cellular structures in super-resolution with SIM, STED and Localisation Microscopy: A practical comparison. *Sci. Rep.* **6**, 27290; doi: 10.1038/srep27290 (2016).

## Supplementary Material

Supplementary Information

## Figures and Tables

**Table 1:** Signal to noise ratios for the three super-resolution techniques.

	Microtubules		
	SIM	STED	SMLM
Mean signal ± standard deviation	7820 ± 2850	8.2 ± 5.2	34687 ± 13329
Mean signal/standard deviation	3.9	2.0	3.4
Mean signal/mean background	54.1	11.7	2075
Total microtubule length (μm)	72	48	70
Total background area (μm^2^)	41	20	69
	**Centrioles**		
	**SIM**	**STED**	**SMLM**
Mean signal ± standard deviation	26129 ± 7252	11.6 ± 2.5	21895 ± 3961
Mean signal/standard deviation	4.2	2.8	2.4
Mean signal/mean background	215	38.0	83.5
Total centriole wall length (μm)	93	91	75
Total background area (μm^2^)	130	89	65

The signal to noise ratio along the microtubules and centriole walls is given as mean signal/standard deviation. The signal to background noise ratio is given as mean signal/mean background.

**Table 2:**
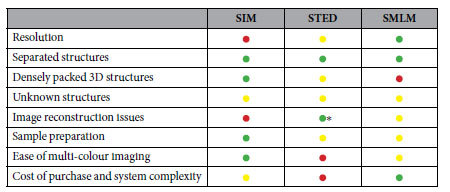
Comparison of the three super-resolution techniques.

Green, good; yellow, medium; red, problematic. *The resolution gain in STED is achieved by optical methods so no reconstruction is strictly needed, however use of image contrast enhancement techniques such as deconvolution can introduce artefacts.
