# Supplementary material for: Imaging cellular structures in super-resolution with SIM, STED and Localisation Microscopy: A practical comparison
Source: Sci Rep. 2016 Jun 6;6:27290. doi: 10.1038/srep27290 (PMC4893670; doi:10.1038/srep27290)
Supplement: Supplementary Information [file srep27290-s1.doc]

**Imaging cellular structures in super-resolution with SIM, STED and Localisation Microscopy: A practical comparison**

Eva Wegel^1,6^, Antonia Göhler^1^, B. Christoffer Lagerholm^2^, Alan Wainman^1,3^, Stephan Uphoff^4^, Rainer Kaufmann^1,5^, Ian Dobbie^1^

^1^Micron Oxford Advanced Imaging Unit, Department of Biochemistry, University of Oxford, South Parks Road, Oxford OX1 3QU, United Kingdom

^2^Wolfson Imaging Centre Oxford, Weatherall Institute of Molecular Medicine, University of Oxford, Headley Way, Oxford OX3 9DS, United Kingdom

^3^Sir William Dunn School of Pathology, University of Oxford, South Parks Road, Oxford OX1 3RF, United Kingdom

^4^Department of Biochemistry, University of Oxford, South Parks Road, Oxford OX1 3QU, United Kingdom

^5^Division of Structural Biology, Wellcome Trust Centre for Human Genetics, University of Oxford, Roosevelt Drive, Oxford OX3 7BN, United Kingdom

^6^Current address: John Innes Centre, Colney Lane, Norwich NR4 7UH, United Kingdom

**Supplementary Information**


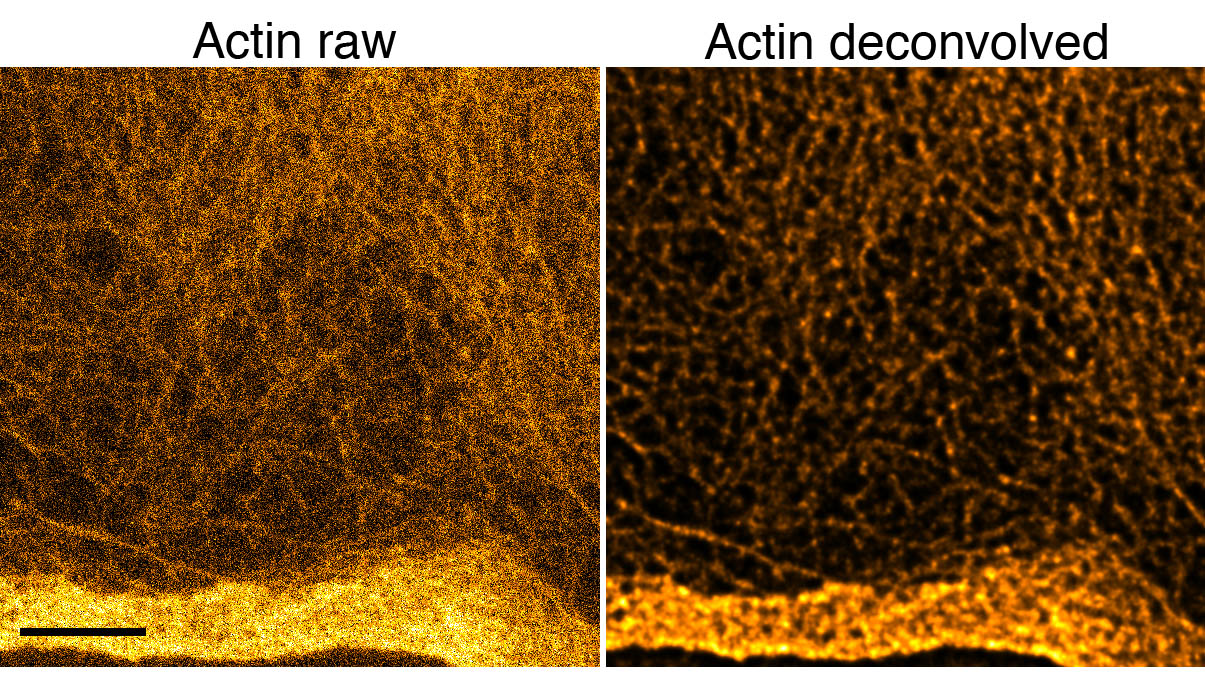


**Supplementary Figure S1**: Comparison of the raw and the deconvolved STED image of the actin meshwork shown in Figure 3. The raw image is very grainy with low contrast. The meshwork is much better resolved in the deconvolved image. Scale bar, 2 µm.


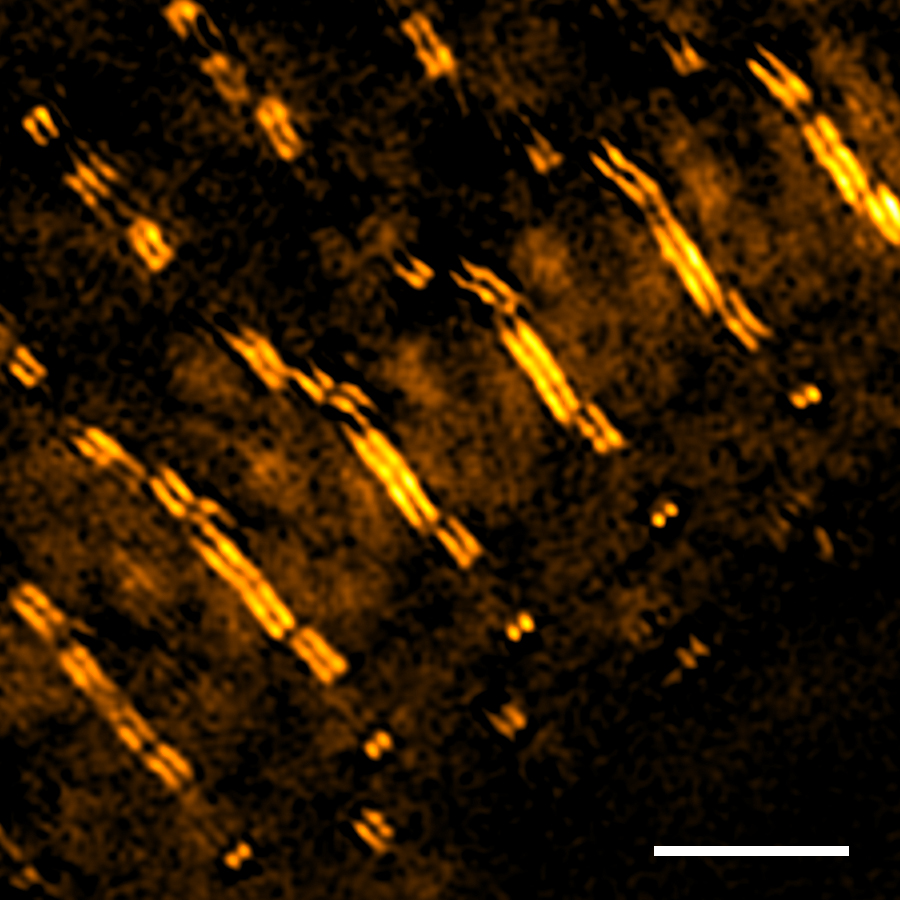


**Supplementary Figure S2**: Example of ‘wiggly’ artificial structures after SIM reconstruction. Titin detection using a primary anti-Titin antibody and a secondary antibody coupled to Alexa Fluor 488 in adult cardiomyocytes. The slide was kindly provided by Katja Gemlich, Dept. of Cardiovascular Medicine, University of Oxford and prepared according to ^1^. All fluorescence signal outside the Titin specific stripes is a reconstruction artefact. The Wiener filter of 0.002 here is too low for a relatively thick sample with low contrast. Scale bar, 2 µm.


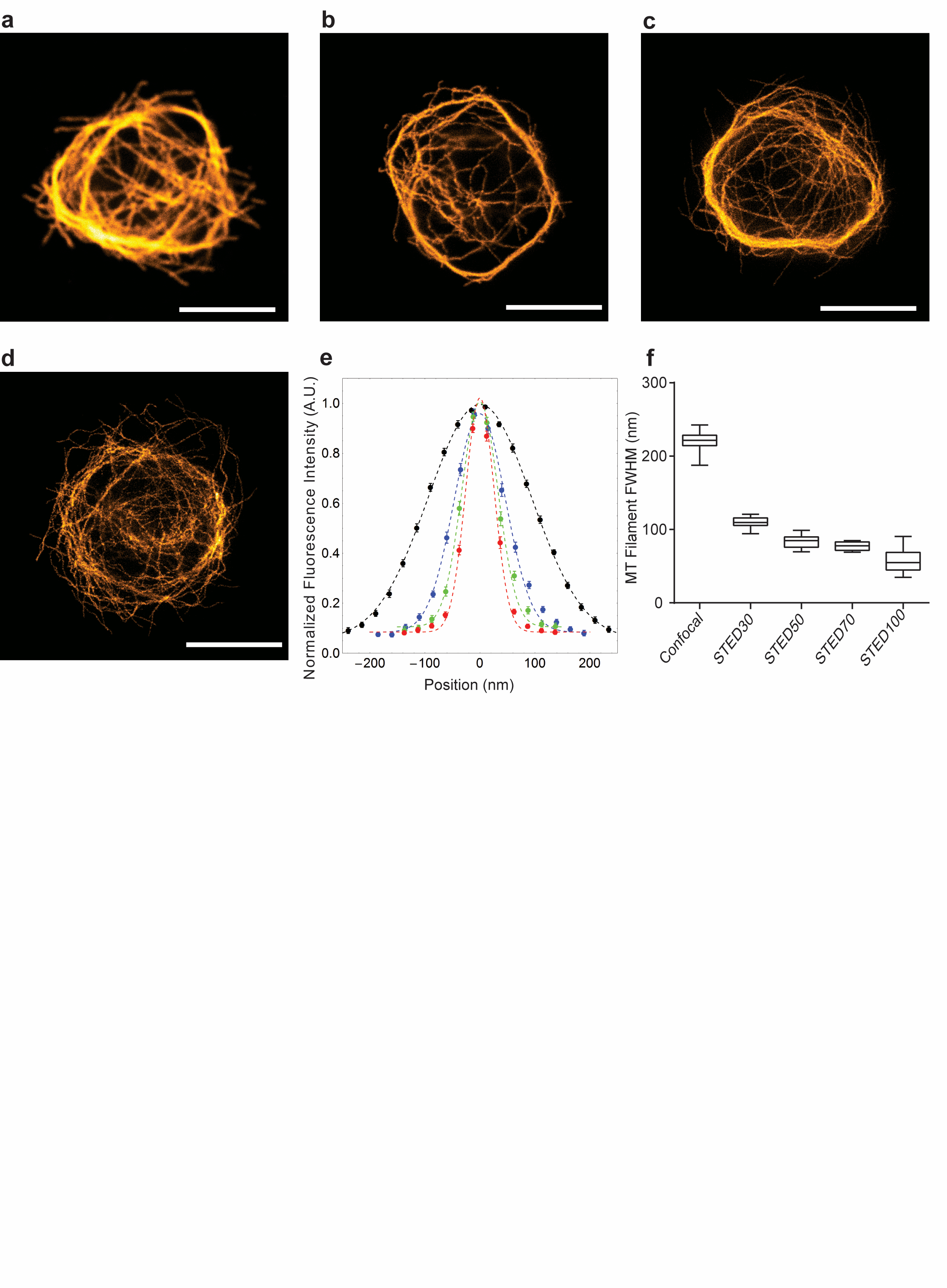


**Supplementary Figure S3:** Dependence of the full width at half maximum (FWHM) of microtubule (MT) filaments on STED laser power. (a) Confocal image. (b) Gated STED image acquired at 30% of maximum STED laser power (P_STED_≈100 mW). (c) Gated STED image acquired at 70% of maximum STED laser power (P_STED_≈250 mW). (d) Gated STED image acquired at 100% of maximum STED laser power (P_STED_≈350 mW). (e) Mean values (± s.e.m.) of intensity line profiles drawn perpendicularly to the microtubule direction in confocal images (Black, N=20), 30% gSTED images (Blue, N=20), 70% gSTED images (Green, N=20), and 100% gSTED images (Red, N=60). (f) Box and Whisker plots of fitted FWHM from intensity line profiles drawn perpendicularly to the microtubule direction in confocal images (N=20), 30% gSTED images (N=20), 50% gSTED images (N=20), 70% gSTED images (Green, N=20), and 100% gSTED images (Red, N=60). These data indicate that the lateral resolution of the STED measurements at 30% of maximum power with the 592 nm STED laser, a power which corresponds to a typical power of 90 mW at back aperture of the objective, results in an equivalent resolution to the SIM measurements in Figure 1 (P-value = 0.076). Scale bars, 5 µm.

**
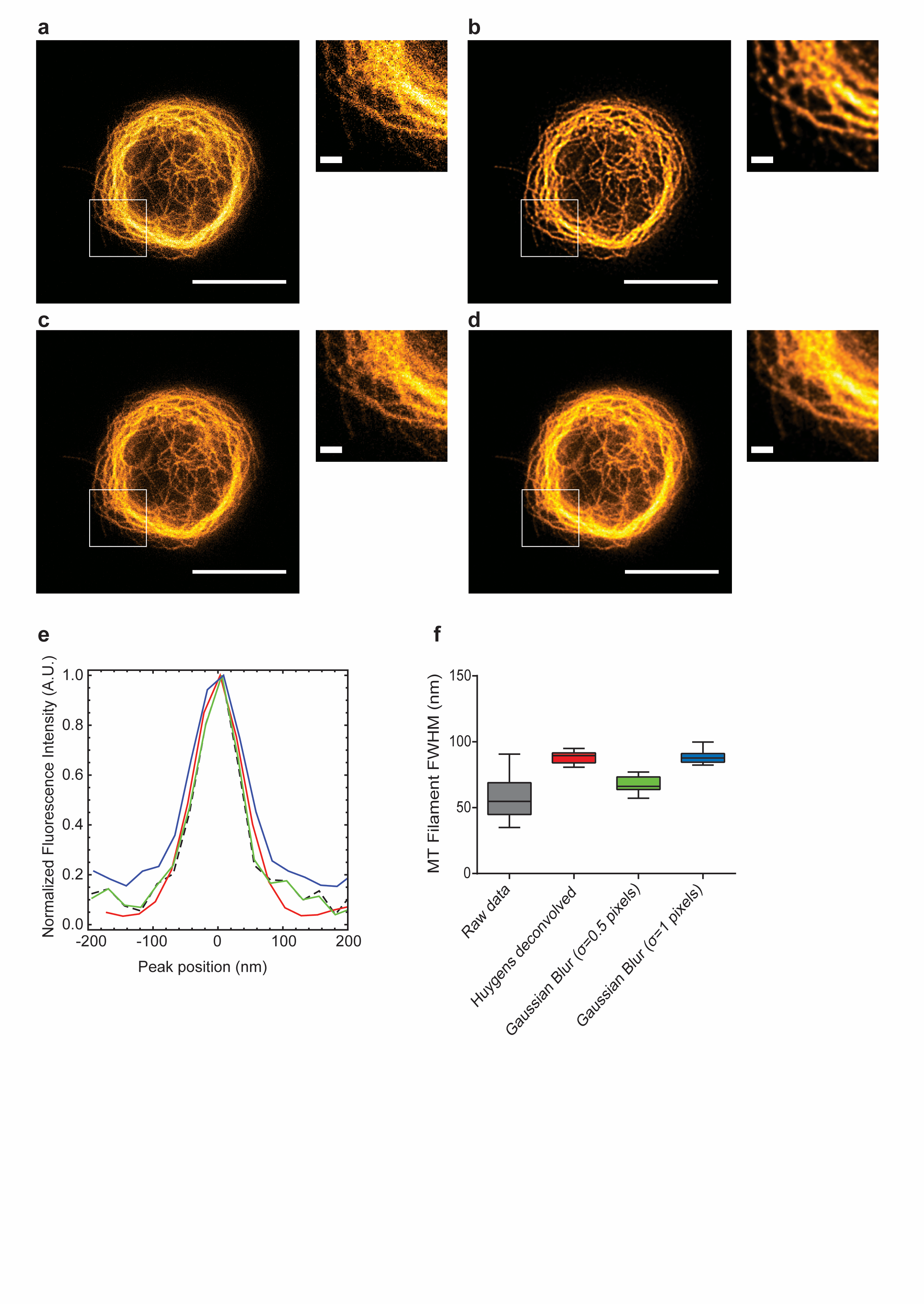
Supplementary Figure S4:** gSTED images typically suffer from poor contrast as a consequence of two factors: a) the photon signal originates from fewer and fewer molecules with increasing STED laser power and b) the detection is time-gated such that only photons that originate from the zero-intensity focus of the gSTED laser in a given time interval (here 1.5 ≤ t ≤ 6.5 ns) after the laser excitation pulse are collected. This problem can be partly remedied by using line averaging in acquisition, by using image post-processing including e.g. deconvolution or Gaussian de-blurring, or by a combination of both. Shown are the effects of different contrast enhancement regimes on image resolution. (a) Representative raw gSTED image of immunolabelled microtubule filaments in Drosophila macrophages acquired at 100% of maximum STED laser power (P_STED_≈300 mW) with a line averaging setting of four. (b) Image after post-processing using Huygens Deconvolution. (c) Image after post-processing using the Gaussian Blur filter in ImageJ with a radius, r, of 0.5 pixels. (d) Image after post-processed using the Gaussian Blur filter in ImageJ with a radius, r, of 1 pixel. Scale bars in full-size images a-d are 5 μm and scale bars in zoomed-in insets are 1 μm. (e, f) Results of quantitative analysis of the effect of image post-processing algorithms on the FWHM of microtubules (N=20) for each condition in (a-d; raw gSTED (black); deconvolved gSTED (Red); Gaussian de-blurring (r=0.5 pixels; green), and Gaussian de-blurring (r=1 pixel; blue). Line profiles (line width of ≈160 nm) were drawn perpendicularly to the direction of the MT filament. (e) Representative normalised line profiles for each condition in (a-d). (f) Results of FWHM measurements of 20 microtubules for each condition.

Each post-processing approach enhances the contrast of the raw image and deconvolution achieves the greatest improvement. The quantitative analysis in (f) shows, however, that all three contrast enhancements increase the FWHM of microtubules.


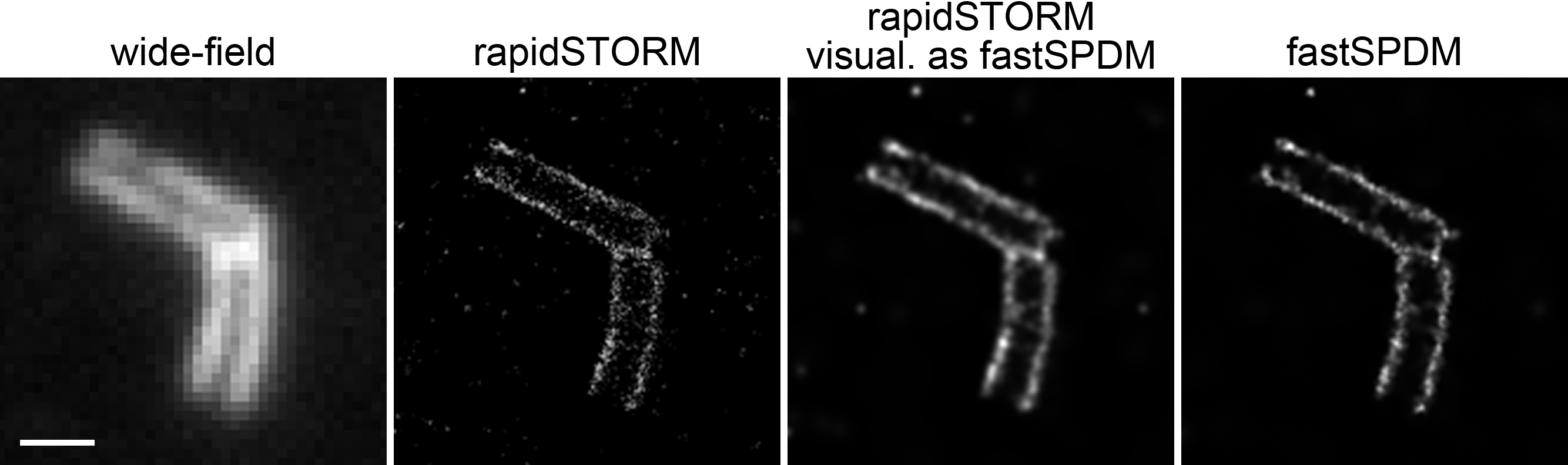


**Supplementary Figure S5**: Comparison of fastSPDM and rapidSTORM for SMLM imaging of centrioles. The same raw data were reconstructed with two different algorithms resulting in differences in background noise level, false positives and structural resolution. With a sliding window background correction function, the fastSPDM software deals better with background suppression ^2^. For better comparison, the position data of rapidSTORM was visualized (in addition to the standard output ^3,4^) with the same method as fastSPDM, which is based on nearest neighbour distances and represents the structural/Nyquist resolution ^5,6^. Scale bar, 500 nm.


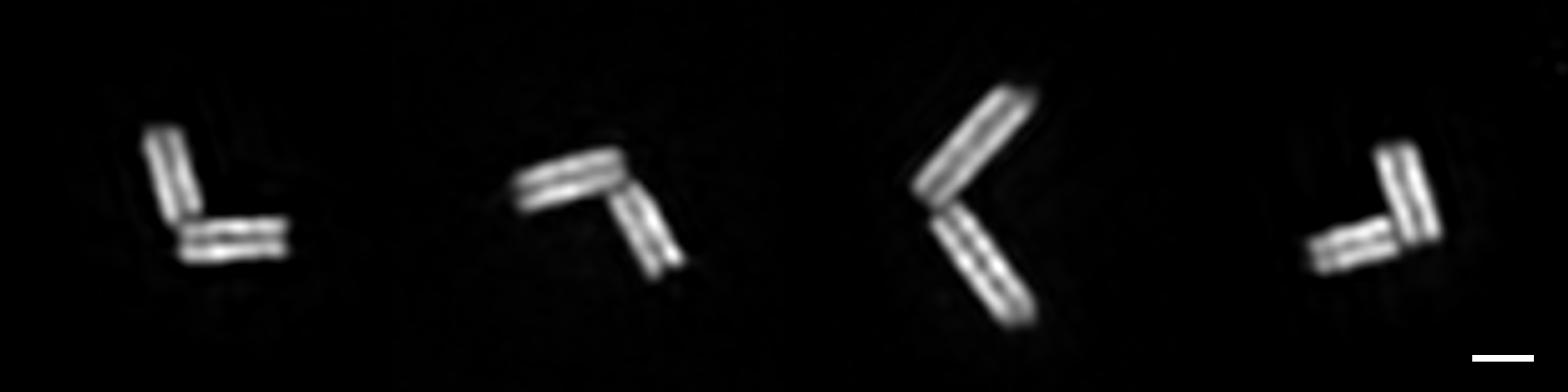


**Supplementary Figure S6**: Representative live SIM images of Asl-GFP. Localisation of Asl in live primary spermatocytes is very similar to that in fixed cells. The gap between the centriole walls is narrower in the live images than in Figure 2 as the C-terminus of the protein is tagged with GFP ^7^ while the Asl antibody recognises the N-terminus. Primary spermatocytes from Drosophila testes were prepared according to ^8^. Shown are single optical sections through the centre of the centriole pairs. Scale bar, 0.5 µm.

**dSTORM microscope setup**

The TIRF microscope for STORM imaging was a custom configuration of ASI Modular Infinity Microscope components (MIM and RAMM, ASI) equipped with an EMCCD camera (iXon 897 Ultra, Andor) and a 100x NA 1.40 oil immersion objective (UPLSAPO, Olympus). A multi laser engine (iChrome, Toptica) with 405 nm (100 mW), 488 nm (100 mW), 561 nm (100 mW), and 640 nm (70 mW) lasers (powers at fiber output) served as excitation light source.

The optical configuration is schematically shown in Fig. 1. The divergent laser beam exiting the optical fiber was collimated using a 150 mm visible achromatic lens (Thorlabs) and focused into the objective back-focal plane using a 200 mm visible achromatic lens (Thorlabs). The fibre mount and lenses were placed on a linear translation mount (Thorlabs) to adjust the angle of excitation. TIRF excitation occurs at excitation angles exceeding a critical angle defined by the refractive index change at the glass-water interphase of the sample. Excitation and emission light were split using a multi-band dichroic mirror (zt405/488/561/640rpc, Chroma) and fluorescence emission filter (zet405/488/561/640m, Chroma), which were supplied pre-aligned in a TIRF filtercube (91041, Olympus). A 300 mm achromatic tube lens module (ASI) focused the fluorescence image onto the camera chip at a total magnification of 167x, giving an image pixel size of 96 nm.

Samples were mounted on a motorized XY stage (MS2000 closed-loop with linear encoders, ASI) with a Z-piezo insert (PZ-2150, ASI), operated by a stage and focus controller (ASI). The objective was attached to a linear translation stage (LS50, ASI) for coarse focusing. Optional focus stabilization was provided by an autofocus system with an 890 nm IR beam (CRISP, ASI), coupled into the microscope using a longpass dichroic mirror (T800lpxrxt, Chroma). For brightfield imaging, a transillumination kit with an Olympus IX2 condenser and LED light source (ASI) was used.

Laser excitation and image acquisition were controlled by Toptica iChrome and Andor Solis software, respectively.


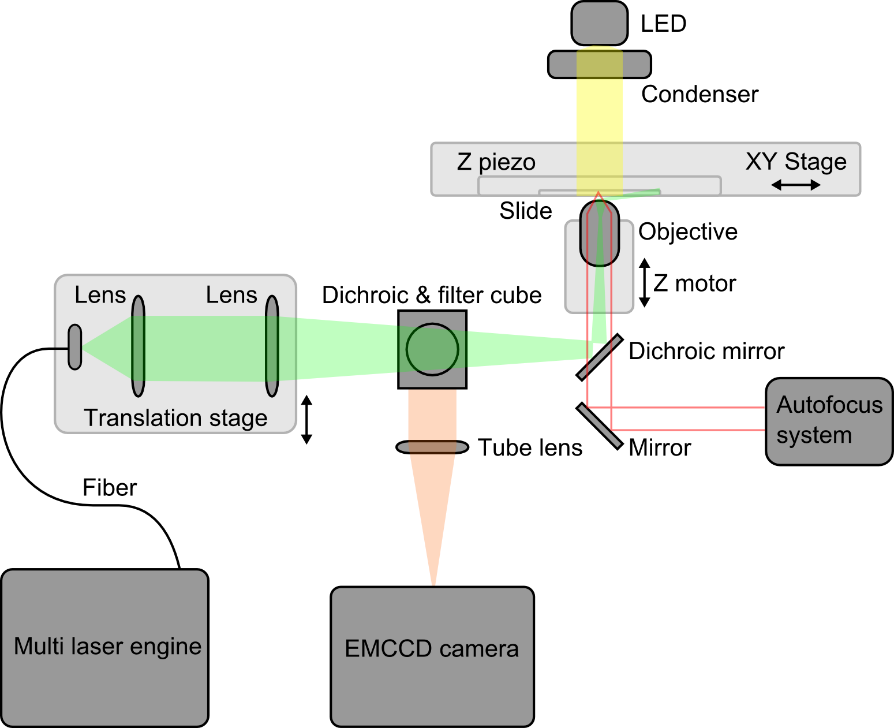


TIRF microscope schematic (not to scale)

References

1 Kotter, S. *et al.* Human myocytes are protected from titin aggregation-induced stiffening by small heat shock proteins. *J Cell Biol* **204**, 187-202, (2014).

2 Grüll, F., Kirchgessner, M., Kaufmann, R., Hausmann, M. & Kebschull, U. in *IEEE International Conference on Field Programmable Logic and Applications.* 1-5.

3 Kaufmann, R. *et al.* Visualization and quantitative analysis of reconstituted tight junctions using localization microscopy. *PLoS One* **7**, e31128, (2012).

4 Johnson, E. *et al.* Correlative in-resin super-resolution and electron microscopy using standard fluorescent proteins. *Sci Rep* **5**, 9583, (2015).

5 Wolter, S. *et al.* Real-time computation of subdiffraction-resolution fluorescence images. *J Microsc* **237**, 12-22, (2010).

6 Wolter, S. *et al.* rapidSTORM: accurate, fast open-source software for localization microscopy. *Nat Methods* **9**, 1040-1041, (2012).

7 Novak, Z. A., Conduit, P. T., Wainman, A. & Raff, J. W. Asterless licenses daughter centrioles to duplicate for the first time in Drosophila embryos. *Curr Biol* **24**, 1276-1282, (2014).

8 Savoian, M. S., Goldberg, M. L. & Rieder, C. L. The rate of poleward chromosome motion is attenuated in Drosophila zw10 and rod mutants. *Nat Cell Biol* **2**, 948-952, (2000).
